# Intermittent Exposure to Xenon Protects against Gentamicin-Induced Nephrotoxicity

**DOI:** 10.1371/journal.pone.0064329

**Published:** 2013-05-30

**Authors:** Ping Jia, Jie Teng, Jianzhou Zou, Yi Fang, Suhua Jiang, Xiaofang Yu, Alison J. Kriegel, Mingyu Liang, Xiaoqiang Ding

**Affiliations:** 1 Division of Nephrology, Zhongshan Hospital Fudan University, Shanghai, China; 2 Department of Physiology, Medical College of Wisconsin, Milwaukee, Wisconsin, United States of America; University of Sao Paulo Medical School, Brazil

## Abstract

Aminoglycoside antibiotics, especially gentamicin, are widely used to treat Gram-negative infections due to their efficacy and low cost. Nevertheless the use of gentamicin is limited by its major side effect, nephrotoxicity. Xenon (Xe) provided substantial organoprotective effects in acute injury of the brain and the heart and protected against renal ischemic-reperfusion injury. In this study, we investigated whether xenon could protect against gentamicin-induced nephrotoxicity. Male Wistar rats were intermittently exposed to either 70% xenon or 70% nitrogen (N_2_) balanced with 30% oxygen before and during gentamicin administration at a dose of 100 mg/kg for 7 days to model gentamicin-induced kidney injury. We observed that intermittent exposure to Xe provided morphological and functional renoprotection, which was characterized by attenuation of renal tubular damage, apoptosis, and oxidative stress, but not a reduction in inflammation. We also found that Xe pretreatment upregulated hypoxia-inducible factor 2α (HIF-2α) and its downstream effector vascular endothelial growth factor, but not HIF-1α. With regard to the three HIF prolyl hydroxylases, Xe pretreatment upregulated prolyl hydroxylase domain-containing protein-2 (PHD2), suppressed PHD1, and had no influence on PHD3 in the rat kidneys. Pretreatment with Xe also increased the expression of miR-21, a microRNA known to have anti-apoptotic effects. These results support Xe renoprotection against gentamicin-induced nephrotoxicity.

## Introduction

Drug nephrotoxicity is one of the most common causes of acute kidney injury (AKI) [1], characterized by an increase in serum creatinine and urea levels and mild to severe proximal renal tubular damage. It occurs in 10–25% of patients treated with the therapeutic doses of aminoglycosides [2], especially gentamicin.

An increasing body of evidence indicates that the mechanisms involved with gentamicin-induced nephrotoxicity are multifaceted [2], [3]. The phenotypic alterations that contribute to acute kidney injury include hemodynamic changes [4], necrosis and apoptosis of renal tubular epithelial cells [5], [6], inflammatory response [7], [8] and oxidative stress [9], In spite of the undesirable toxic effects of gentamicin, it is still widely used against infections by Gram-positive and Gram-negative aerobic bacteria due to its excellent antibacterial profile and efficacy [10], [11]. Given the largely ineffective therapeutic strategies for preventing the incidence of gentamicin-induced nephrotoxicity in clinic, the development of new and efficient therapeutic interventions is clearly needed.

The noble gas xenon (Xe) has been used as an anesthetic for more than 50 years. Xe has many of the properties of the ideal anesthetic including a small blood-gas partition coefficient [12] and low toxicity. Moreover, Xe is not an environmental pollutant [13], and does not induce significant alterations of the hemodynamic and cardiovascular patterns [14], [15]. Interestingly, many studies have demonstrated that Xe also provided substantial organoprotective effects in acute injury of the brain [16], [17], heart [18]–[20], and kidney [21], independent of anesthesia.

The precise mechanisms underlying the protective effects of Xe remain to be elucidated. Ma et al. [21] demonstrated that Xe preconditioning protected against renal ischemic-reperfusion injury (IRI) in mice through activation of the mammalian target of rapamycin (mTOR) pathway and subsequent increased expression of HIF-1α and its downstream effectors. Here we demonstrated that intermittent exposure to Xe protected rats against functional and morphological indices of gentamicin-induced nephrotoxicity. The renopretection provided by Xe was characterized by attenuation of tubular damage, apoptosis, and oxidative stress, but not inflammation. The possible mechanisms for this protection include up-regulation of miR-21, hypoxia-inducible factor 2α (HIF-2α) and its downstream effector vascular endothelial growth factor (VEGF).

## Materials and Methods

### Animals

Experiments were performed with male Wistar rats (Animal Center of Fudan University, Shanghai, China), weighing 200–230 g, housed in temperature- and humidity-controlled cages (20°C and 60% humidity) with free access to water and rodent food which were obtained from Animal Center of Fudan University, and a 12-h light/dark cycle. All protocols were approved by the Institutional Animal Care and Use Committee of Fudan University. All surgery was performed under sodium pentobarbital anesthesia at a dose of 40 mg/kg, and all efforts were made to minimize suffering, such as improving the quality of anaesthetic practices and investigating ways to reduce the risk of infections.

### Experimental Protocol 1 (Figure S1A)

Rats were randomly assigned into four groups: (i) control group: rats received a daily intraperitoneal (i.p.) injection of 1 ml saline for 7 days; (ii): gentamicin group (gent) : received a daily i.p. injection of gentamicin sulfate solution (Tianjin Pharmaceutical Company, China) at a dose of 100 mg/kg/BW for 7 days, which is well known to cause significant nephrotoxicity in rats [22], [23]; (iii) N_2_+gentamicin group (N_2_+gent) : rats pretreated with 70% nitrogen balanced with 30% oxygen for 2 hrs, followed by 7 days of gentamicin treatment initiated 24 hrs later, and repeatedly treated with 70% nitrogen for 30 minutes on day 2, 4, and 6 during the 7-day administration of gentamicin; (iv) Xe+gentamicin group (Xe+gent) : rats treated with 70% xenon (balanced with 30% oxygen) intermittently before and during the gentamicin administration, similar to the N_2_+gentamicin group. On the 2^nd^, 4th and 7th day, rats were placed in metabolic cages individually for 24 h and urine was collected for determination of creatinine clearance (Ccr) and N-acetyl-β-D-glucosaminidase (NAG). Six rats each group were sacrificed under anesthesia on days 2, 4, and 7 after the last gentamicin injection, respectively. Blood was taken from the abdominal aorta for the assay of creatinine and urea nitrogen. Kidneys were harvested and weighed, One kidney was for the analysis of histology, immunohistochemistry. One kidney was frozen in liquid nitrogen and stored at − 80°C for further assay.

### Experimental Protocol 2 (Figure S1B)

Rats were randomly divided into three groups including six animals each: (i) control: rats with no treatment; (ii) Xe: rats exposed to 70% xenon balanced with 30% oxygen for 2 hrs; (iii) N_2_: rats exposed to 70% N_2_ balanced with 30% oxygen for 2 hrs. Rats were sacrificed 24 hs later**.** Kidneys were rapidly removed for further assay.

### Urinalysis and Blood Chemistry Examination

Urinary N-acetyl-β-D-glucosaminidase (NAG) level was determined by using standard diagnostic kit (Jiancheng Biotech., Nanjing, China) according to the manufacturer’s instructions. Urine creatinine (Ucr), serum creatinine (Scr) and blood urea nitrogen (BUN) levels were measured using an automated analyzer (Vet test 8008, USA). Creatinine clearance (Ccr) was calculated according to the standard formula. Tumor necrosis factor alpha (TNF-α) in serum was determined using commercial enzyme-linked immunosorbent assay (ELISA) kits (R&D Systems, Inc., Minneapolis, USA) according to the manufacturer’s instructions.

### Histopathological Examinations

Kidney slices were fixed in 10% formalin, embedded in paraffin wax, cut into 5 µm sections and stained with hematoxylin and eosin. The tissues were evaluated by light microscopy. Histopathological scoring was performed by a pathologist unaware of the experimental protocol, according to the severity of tubular injury in renal cortex on a semi-quantitative scale [24]: no injury(0), mild: <25% (1), moderate: <50% (2), severe: <75% (3) and very severe: >75% (4). More than 20 consecutive fields were examined under 400× magnification.

### TUNEL Staining

Terminal deoxynucleotidyl transferase-mediated dUTP nick end labeling (TUNEL) method was used to assess apoptosis based on labeling of DNA strand breaks, according to the manufacturer’s protocol (In situ Cell Death Detection kit, POD,Roche). The number of TUNEL-positive cells from 10 areas of randomly selected renal cortex was counted under a 40× objective lens of light microscope.

### Immunohistochemistry

Immunohistochemical staining was performed in 4 µm paraffinized sections. After dewaxed and dehydrated, the sections were incubated with 3% H_2_O_2_ for 10 min to eliminate endogenous peroxidase activity and were treated with normal goat serum for 20 min. Subsequently, they were incubated at 4°C overnight with primary antibodies against ED-1 (rabbit monoclonal, 1∶100, Novus, USA) or TNF-α (rabbit polyclonal, 1∶2000, Abcam, USA). Then the sections were incubated with horseradish peroxidase-conjugated secondary antibody (anti-rabbit IgG). After rinsing with PBS, the sections were stained with 3,3′-diaminobenzidine (Sigma), then counterstained with hematoxylin and evaluated under light microscopy.

### Determination of Lipid Peroxide

Malondialdehyde (MDA), as a naturally occurring product of lipid peroxidation, was determined using supernatant of the renal cortical homogenate, according to the manufacturer’s protocol (TBARS Assay Kit, Cayman Chemical Company, USA).

### Real-Time Reverse Transcription–PCR

Total RNA from kidney tissue was extracted using Trizol (Invitrogen), followed by quantification, and then reverse-transcribed (PrimeScript® RT reagent Kit, TaKaRa, Japan). The cDNA product was used for real-time PCR (SYBR® Premix Ex Taq™ TaKaRa, Japan). The sequences of PCR primers (Sangon, shanghai, China) were as follows: HIF-1α forward 5′- CACTGC -ACAGGCCACATTCAT-3′, reverse 5′- AAGCAGGTCATAGGCGGTTTC-3′; HIF-2α forward 5′-GTCACCAGAACTTG TGC-3′, reverse 5′-CAAAGATGCTGTT-3′; VEGF forward 5′-CAGCTATTGCCG TCCAATTGCA -TGGA-3′, reverse 5′- CCAGGGCTTCATCATTGCA-3′; PHD1 forward 5′-GTGGCGAGGCAAGTTCTAGGC-3′, reverse 5′-CCTGCACAGTGGC GGATTAC-3′; PHD2 forward 5′- CCATGGTCGCCTGTTACCC-3′, reverse 5′- CGTACCTTGTGGCGTATGCAG-3′; PHD3 forward 5′- CCTGGTCTGATAGGA GGAGGTTT-3′, reverse 5′- GCCCTTTCTAGAGGCGATAATGT -3′; β-actin forward 5′- GATTACTGCCCTGGCTCCTA -3′,reverse 5′- TCATCGTACTCCTGC TTGCT-3′. Expression level of miR-21 was quantified using real-time reverse transcription**-**PCR with the Taqman chemistry (Applied Biosystems) as described previously [25]. 5S ribosomal RNA and β-actin mRNA were used as endogenous control for miRNAs and mRNAs, respectively. The relative gene expressions were calculated in accordance with the ΔΔCt method. Relative mRNA levels were expressed as 2^−ΔΔCt^ (Tn) and ratios to control.

### Preparation of Homogenates and Western Blot Analysis

The dissected renal cortices were homogenized in ice-cold lysis buffer (25 mmol/l Na-HEPES, 1 mmol/l EDTA, and 0.1 mmol/l PMSF). After being centrifuged at 10,000 g for 5 min at 4°C, the supernatant was collected. Samples (50 µg/Lane) were loaded and separated by sodium dodecyl sulfate-polyacrylamide gel electrophoresis (SDS-PAGE), and then transferred to polyvinylidine difluoride membranes. The membranes were blocked with 5% non-fat milk and hybridized with primary antibodies against HIF-1α (rabbit polyclonal 1∶500, Abcam, USA), HIF-2α (rabbit polyclonal 1∶1000, Novus, USA), prolyl-4-hydroxylase domain-containing proteins (PHD1, PHD2, PHD3, goat polyclonal, 1∶200, Sant Cruz Biotechnology Inc.), VEGF (polyclonal 1∶1000, Abcam, USA), followed by horseradish peroxidase-conjugated secondary antibodies and visualization with the enhanced chemiluminsecense system. Results were normalized with α-tubulin or GAPDH and expressed as ratios to control.

### Statistical Analysis

Statistical analysis was performed using the statistical software SPSS Version 16.0. Data were presented as means ± S.E., Differences between multiple-group means were evaluated by one-way ANOVA. A p-value <0.05 was considered significant.

## Results

### Xe Treatment Protected Renal Function Against Gentamicin Injury

**Figure 1:**
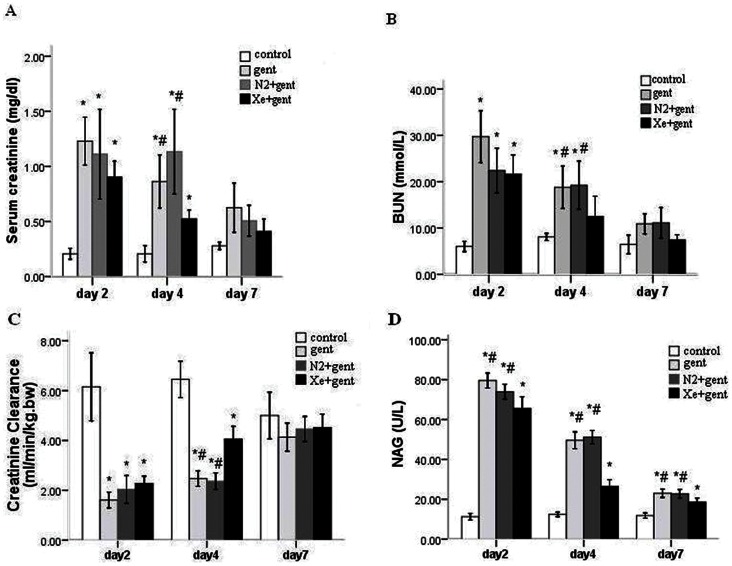
Xenon protected renal function against gentamicin-induced nephrotoxicity. A: serum creatinine concentration (Scr) from all groups 2, 4 and 7 days after 7-day gentamicin or saline injection. B: blood urea nitrogen concentration (BUN). C: creatinine clearance (Ccr). D: Urinary N-acetyl-β-D-glucosaminidase (NAG). Xenon treatment significantly attenuated the elevation of Scr, BUN, NAG and reduction of Ccr 4 days after 7-day gentamicin injection compared with the gentamicin group and N_2_+gentamicin group. *P<0.01 *vs.* control group; #P<0.05 *vs.* Xe+gentamicin group. n = 6/group at each time point.

Animals showed marked deterioration of renal function following 7 days of daily gentamicin injections. This was characterized by significant increases in serum creatinine, blood urea nitrogen levels and a decrease in creatinine clearance level, which peaked at 48 hrs after the last injection (Scr, 1.42±0.21 mg/dl; BUN, 29.72±6.85 mmol/L; Ccr, 1.61±0.38 ml/min/kg.bw). Animals that underwent intermittent exposure to 70% Xe, balanced with 30% oxygen, exhibited slight, but not significant, reductions in Scr, BUN and an increase in Ccr level 48 hrs after the last injection of gentamicin (Scr, 1.00±0.11 mg/dl; BUN, 21.59±5.11 mmol/L; Ccr, 2.28±0.36 ml/min/kg.bw). At 4 days after the final gentamicin administration, there were significant improvements in renal functional parameters in the Xe+gentamicin group when compared with the gentamicin or N_2_+gentamicin control groups (Scr, 0.59±0.07 mg/dl *vs*. 1.18±0.27 mg/dl and 0.80±0.11 mg/dl; BUN, 12.47±5.40 mmol/L *vs.* 18.80±5.62 mmol/L and 24.25±13.21 mmol/L; Ccr, 4.06±0.62 ml/min/kg.bw *vs.* 2.47±0.38 ml/min/kg.bw and 2.37±0.40 ml/min/kg.bw, p<0.05) (Figure 1 A, B and C). Treatment with 70% N_2_ produced no significant reduction in kidney injury compared with the gentamicin-treated group.

NAG had higher activities in the urine of gentamicin administered animals 2 and 4 days after the final injection compared to the control (P<0.01). The enzyme activities were significantly reduced in Xe treatment group when compared with the gentamicin group and N_2_ treatment group at the two time points (P<0.05) (Figure 1 D).

### Functional Protection was Accompanied by Attenuation of Tubular Damage, Apoptosis, and Oxidative Stress, but not Inflammation

**Figure 2:**
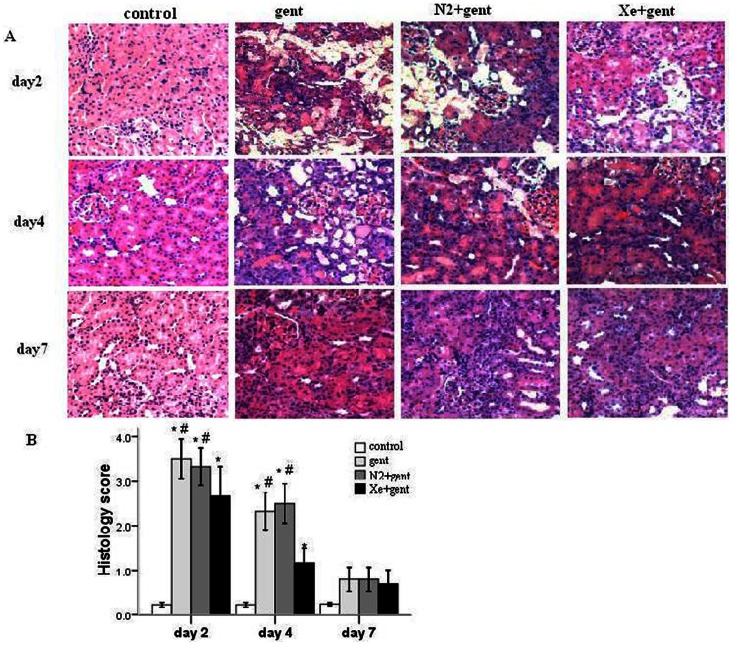
Effect of xenon on renal morphology. A: representative images of the cortex of renal sections from all groups 2, 4 and 7 days after 7-day gentamicin or saline injection respectively. (hematoxylin-eosin staining, 100×).B: renal tubular injury assessed by histology grading scores in all groups. Xenon treatment significantly attenuated the renal tubular injury 2 and 4 days after 7-day gentamicin injection compared with the gentamicin group and N_2_+gentamicin group. *P<0.01 vs control group; #P<0.05 *vs.* Xe+gentamicin group, n = 6/group at each time point.

Parallel to the deterioration of functional parameters, severe morphological damage occurred in the kidneys of rats in the gentamicin and N_2_+gentamicin groups, especially 48 hrs after the final gentamicin injection. The structural derangements were most prominent in the cortical proximal segments, and included massive tubular epithelial cell degeneration, desquamation and necrosis, naked basement membranes, proteinaceous or cellular casts, and inflammatory cell infiltration. In contrast, animals treated with Xe showed mild to moderate tubular epithelial changes, such as focal vacuolization, cytoplasmic granularity and sporadic cell necrosis (Figure 2A).

Morphologic evidence of gentamicin-induced nephrotoxicity was assessed using a histopathologic scoring of cortical tubular damage, which integrated tubular cell necrosis or swelling, tubular casts, brush border loss and interstitial inflammatory cell infiltration in 10 non-overlapping fields (20×magnification) of the cortex. The histologic score in the gentamicin group at the 48 hr time point was 3.50±0.22, which decreased significantly to 2.67±0.33 (*P*<0.05) in the Xe treatment group. A similar change also occurred at the 4-day time point, which paralleled the improvement of renal function. The N_2_ treatment group showed no significant difference from gentamicin-treated group in histologic score (Figure 2B).

**Figure 3:**
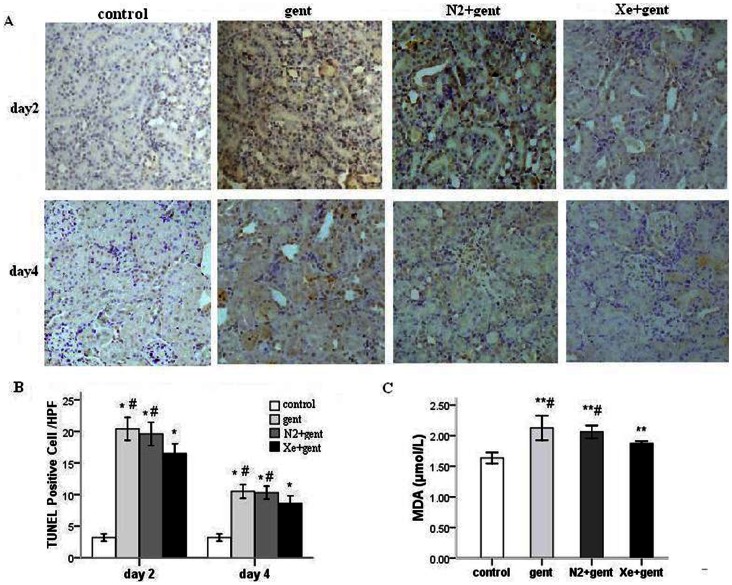
Xenon treatment significantly attenuated cell apoptosis and decreased malondialdehyde (MDA) in the kidney. A: TUNEL-positive cells in renal sections from all groups 2 and 4 days after 7-day gentamicin injection. B: mean value of TUNEL-positive cells in renal sections from all groups. C: MDA concentration in renal tissue 4 days after 7-day gentamicin injection. *P<0.01, **P<0.05 *vs.* control group; #P<0.05 *vs.* Xe+gentamicin group, n = 6/group at each time point.

To estimate renal injury at cellular level, terminal deoxynucleotidyl transferase–mediated digoxigenin deoxyuridine nick-end labeling (TUNEL) staining was used to analyze apoptosis of renal cells. TUNEL-positive cells were prominent in renal proximal tubules (Figure 3A), which were significantly fewer in the Xe+gentamicin group (16.50±0.76) than the gentamicin group (20.40±0.91) in kidneys taken 2 days after the final injection (*P*<0.05). Similar differences were also observed at the 4-day time point (Figure 3 B). The N_2_ treatment group showed no significant difference from gentamicin-treated group in apoptosis at these two time points.

Malondialdehyde (MDA), a naturally occurring product of lipid peroxidation, is used as an indicator of oxidative stress injury [26]. Thiobarbituric Acid Reactive Substances (TBARS) assay was used to detect MDA content in renal tissues. We found that MDA concentration in renal cortical tissues was significantly reduced in Xe treatment group at 4 day after the last administration of gentamicin compared with the gentamicin group and N_2_ treatment group (1.87±0.02 µmol/L *vs.* 2.13±0.10 µmol/L and 2.06±0.05 µmol/L; P<0.05) (Figure 3 C).

**Figure 4:**
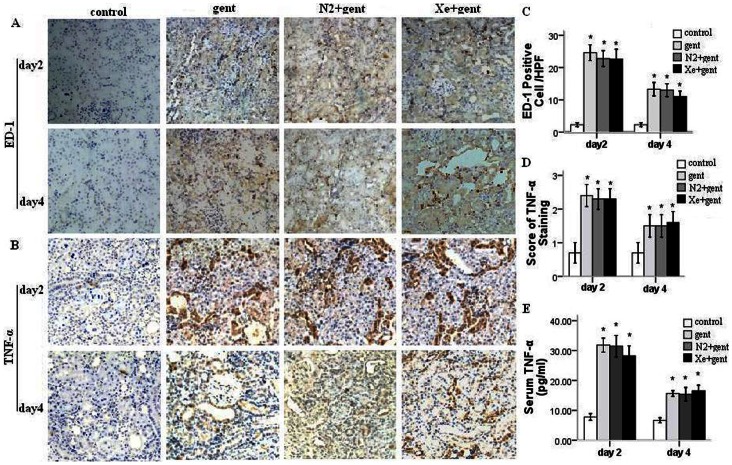
Immunohistochemical staining of ED-1 and TNF-α in renal sections. Representative photomicrographs, 100×, A–B: brown color indicates positive staining. A: the expression of ED-1 in kidneys 2 and 4 days after 7-day gentamicin injection. B: the expression of TNF-α in kidneys. C: mean value of ED-1 positive cells in renal sections from all groups. D: scores of TNF-α staining-positive areae in renal sections from all groups. E: Serum TNF-α level. There were no significant difference in expression of ED-1 and TNF-α in kidney between gentamicin group, N_2_+gentamicin group and Xe+gentamicin group. The level of serum TNF-α was not significantly different between the experimental groups. *P<0.01 *vs.* control group, n = 6/group at each time point.

To explore the effect of Xe treatment on inflammatory response in kidney, we examined the protein expression of a macrophage/monocyte marker (ED-1) and tumor necrosis factor-alpha (TNF-α). Immunohistochemical staining revealed that the expression of TNF-α was mainly localized to the injured tubulointerstitium and ED-1^+^ cells (macrophages) were abundant in peritubular spaces (Figure 4 A and B). Quantitatively, expression of both proteins was significantly higher in three experimental groups compared with control group (P<0.01), whereas there were no significant differences in expression between the Xe+gentamicin, gentamicin, and N_2_+gentamicin treated groups (P>0.05) (Figure 4 C and D). The serum TNF-α concentration was significantly elevated in three experimental groups compared with control group (P<0.01), while there were no significant differences between the Xe+gentamicin, gentamicin, and N_2_+gentamicin treated groups (P>0.05) (Figure 4 E). These results indicated that Xe treatment did not attenuate inflammation in gentamicin-injured kidney.

### Xe Pretreatment Induced Expression of HIF-2α and its Downstream Effector, but did not Affect HIF-1α

**Figure 5:**
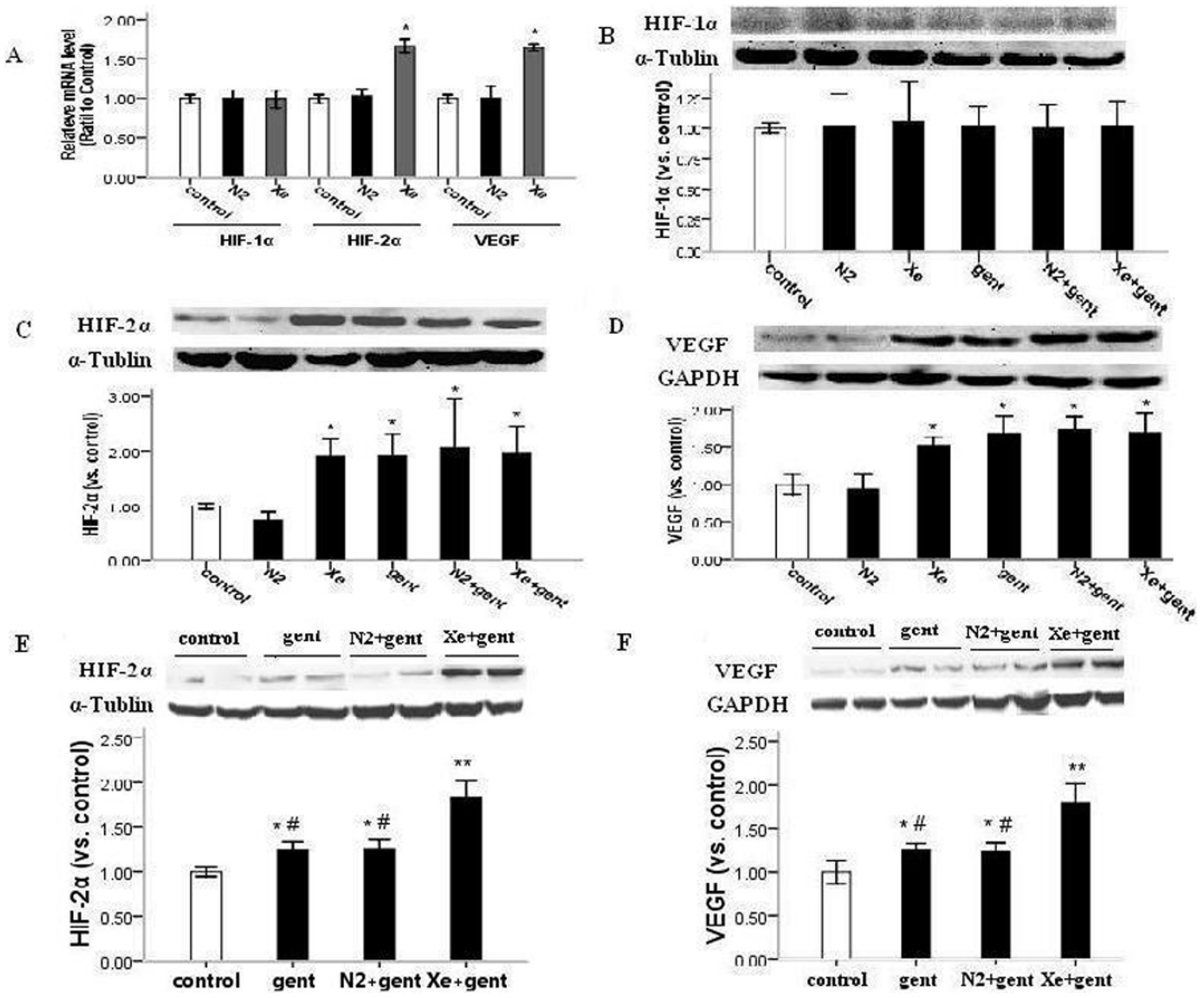
Xe pretreatment up-regulated HIF-2α and VEGF, but not HIF-1α. A: the relative mRNA levels of HIF-1α, HIF-2α and VEGF by real-time reverse transcriptase-PCR analysis from N_2_ group and Xe group 24 hrs after N_2_ or Xe pretreatment alone. Xe upregulated the mRNA level of HIF-2α and VEGF, but not HIF-1α. B–F: representative images of western blot analysis of HIF-1α, HIF-2α and VEGF from N_2_ group and Xe group 24 hrs after N_2_ or Xe pretreatment alone, and from gentamicin group, N_2_+gentamicin group, Xe+gentamicin group and control 48 hrs or 4 days after 7-day gentamicin administration. Relative intensities of HIF-1α, HIF-2α and VEGF blots normalized to control. B: Xe or N_2_ pretreatment or gentamicin administration did not affect HIF-1α. 24 hrs after N_2_ or Xe pretreatment alone or 48 hrs after 7-day gentamicin administration. HIF-1α barely detectable level in all groups. C-D: Xe pretreatment or gentamicin treatment significantly up-regulated HIF-2α and VEGF 24 hrs after Xe pretreatment alone or 48 hrs after 7-day gentamicin administration. E-F: Xe+gentamicin group significantly up-regulated HIF-2α and VEGF 4 days after 7-day gentamicin administration. *P<0.05, **P<0.01 *vs.* control group, #P<0.05 vs Xe+gentamicin group, n = 5–6.

To explore the underlying mechanisms of Xe-induced renoprotection, we employed an additional experiment in which animals were treated with 70% Xe or 70% N_2_ balanced with 30% oxygen for 2 hrs. We then isolated the rat kidneys 24 hours later and measured HIF-1α and HIF-2α expression and a selected downstream effector VEGF. Quantitative RT-PCR analysis of mRNA expression showed that HIF-2α and VEGF were significantly higher in Xe pretreatment group than in the N_2_-pretreatment group and control (P<0.05), while the HIF-1α mRNA expression was not affected (Figure 5A).

As showed in Figure 5, parallel to alteration of the mRNA levels, Xe pretreatment upregulated the protein expression of HIF-2α and VEGF in kidney, but not HIF-1α. We found that gentamicin administration also upregulated HIF-2α and VEGF protein expression, and there were no significant differences in expression 48 hrs after 7-day gentamicin administration between the gentamicin-treated, Xe+gentamicin and N_2_+gentamicin groups. However, at 4 days after the last gentamicin administration, HIF-2α and VEGF were significantly higher in Xe+gentamicin group than in the gentamicin and N_2_+gentamicin groups (P<0.05). In all experimental groups, HIF-1α was unaffected and only present at a barely detectable level.

### Effect of Xe Pretreatment on Expression of PHDs in Kidney

**Figure 6:**
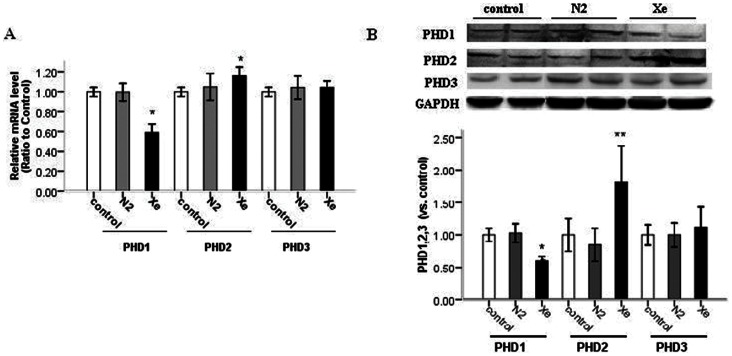
Xe pretreatment up-regulated PHD2, down-regulated PHD1, but did not affect the expression of PHD3. A: the relative mRNA levels of PHD1, PHD2 and PHD3 by real-time reverse transcriptase (RT)-PCR analysis in kidney 24 hrs after 70% N_2_ or 70% Xe pretreatment alone. B: representative images of western blot analysis of PHD1, PHD2 and PHD3 in kidney 24 hrs after N_2_ or Xe pretreatment alone. Relative intensities of PHD1, PHD2 and PHD3 blots normalized to control. *P<0.05 *vs*. control group.

HIF prolyl hydroxylases, PHD1, PHD2, and PHD3, have been reported to contribute to the regulation of both HIF-1α and HIF-2α [27], [28], however the effects of the PHDs on different HIF-α isoforms were not equivalent.^26^ We found that 24 h after exposure to 70% Xe mRNA expression of PHD1 significantly decreased in the renal cortex, while PHD2 significantly increased, and PHD3 was not affected. The PHDs mRNA levels were not significantly different in N_2_-treated group, compared with control (Figure 6 A). Furthermore, the protein expression of PHD1, PHD2, and PHD3 in the cortex paralleled to patterns of the mRNAexpression (Figure 6 B). From the consistent changes in mRNA and protein expression of PHDs in the renal cortex, we could speculate that Xe treatment may regulate PHDs at the transcriptional level.

### Xe Pretreatment Upregulated miR-21

**Figure 7:**
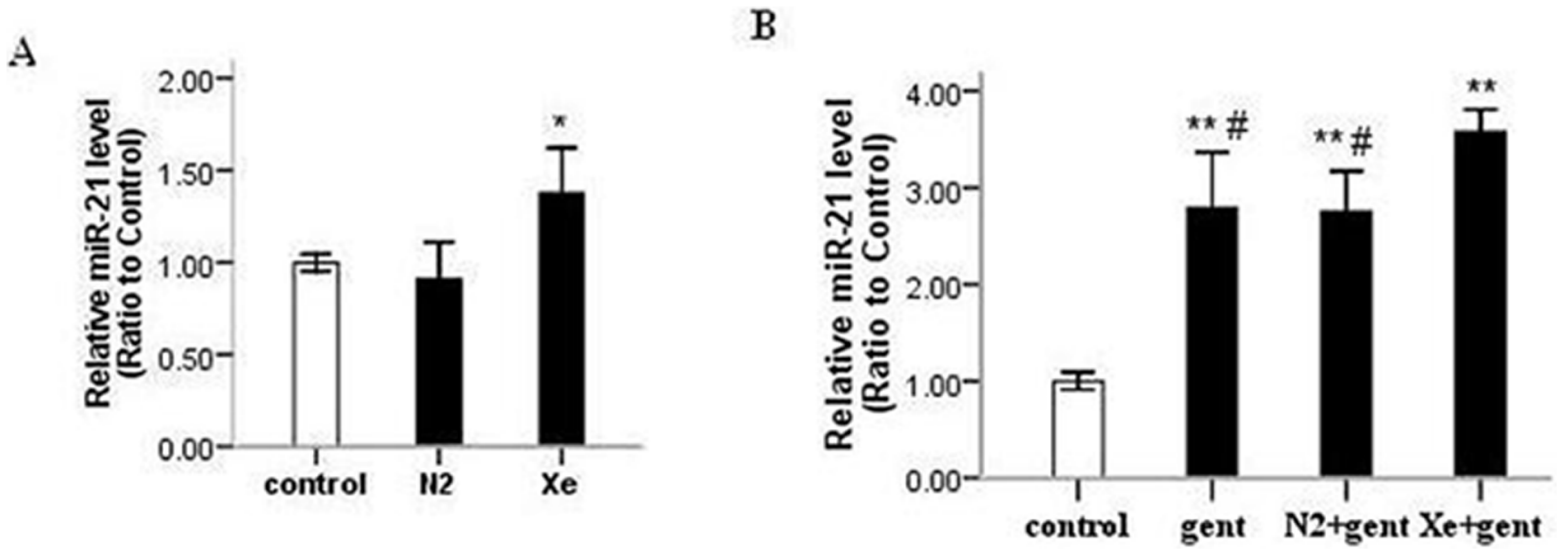
Expression of miR-21 in rat kidneys. A: Xe pretreatment significantly upregulated miR-21 in rat kidneys 24 hrs after Xe pretreatment. B: gentamicin treatment significantly up-regulated miR-21 4 days after 7-day gentamicin administration, which was further increased by Xe. *P<0.05 *vs*. control group, **P<0.01 *vs*. control group, #P<0.05 *vs.* Xe+gentamicin group, n = 5–6.

The role of miRNAs in gentamicin-induced acute kidney injury is not known. It has been shown that miR-21 is a strong anti-apoptotic factor [29], [30]. Given that Xe treatment attenuated tubular cell apoptosis (see above), we measured expression of miR-21 in our renal cortex samples by Taqman real-time PCR. Interestingly, we found that Xe pretreatment significantly upregulated miR-21 expression in adult rat kidney at 24 hr after 70% Xe exposure (P<0.05) (Figure 7 A). We also found that gentamicin treatment activated miR-21 expression at 4 day after 7-day gentamicin administration, and Xe+gentamicin group showed higher miR-21 expression than either the gentamicin or N_2_+gentamicin group (Figure 7 B).

## Discussion

This study shows for the first time that intermittent exposure to Xe could protect against gentamicin-induced nephrotoxicity. To date, most studies on xenon have focused on the effect of pre-conditioning and organoprotection against ischemia-reperfusion injury. In the kidney, Ma et al. [21] demonstrated that Xe preconditioning protected against renal ischemic-reperfusion injury in mice via increased expression of HIF-1α. Rizvi et al. [31] found that 75% Xe had a protective effect on oxygen and glucose deprived human tubular kidney cells by increasing expression of p-Akt, HIF-1α and Bcl-2. The present study was the first to examine the effect of Xe on drug nephrotoxicity, and showed that intermittent exposure to Xe both before and during gentamicin administration provided significant morphological and functional protection against gentamicin-induced nephrotoxicity. This study explores a new idea, and will be a platform for more follow-up basic sciences and clinical studies on this inert gas in the role of renal protection.

Gentamicin injury in the kidney is characterized by renal tubular epithelial cell necrosis, apoptosis, inflammatory response, oxidative stress, and vascular contraction. Dysfunction of mitochondria is one of the earliest indexes of gentamicin-induced nephrotoxicity, which produces excessive ROS, resulting in morphological and functional changes. Resent study demonstrated that application of a mitochondria-targeted antioxidant SkQR1 provided renoprotective effects against gentamicin-induced nephrotoxicity and renal ischemia-reperfusion injury in rats via normalizing the level of ROS and lipid peroxidized products in kidney mitochondria [32], [33]. Silachev et at. [34] demonstrated that both renal ischemic preconditioning and antioxidant SkQR1 could induce the release of EPO from the kidney, and ameliorated brain damage through kidney-to-brain cross-talk. The present study showed that Xe attenuated tubular necrosis, apoptosis, and oxidative stress, but did not affect inflammation. Moreover, we found that both 70% xenon and gentamicin treatment upregulated HIF-2α and its downstream effector VEGF. Xe combined with gentamicin significantly up-regulated HIF-2α and VEGF 4 days after 7-day gentamicin administration. The mechanism underlying upregulation of HIF-2α might be multifold. ROS could be an additional factor that induces expression of HIF and activate protective pathways. It appears that the renoprotective effect of Xe against gentamicin injury might involve up-regulation of HIF-2α and its downstream effectors, such as VEGF and EPO.

Expression of HIF1-α and HIF2-α can be induced in the kidney differentially depending on the type of stimulus, renal cell type and kidney zone [35], [36]. HIF-2α upregulates expression of many genes involved in erythropoiesis, glycolysis, and angiogenesis, like VEGF [37] and EPO [38]–[40], which are important for maintenance of normal kidney functions. Kojima et al. [41] demonstrated that HIF-2α conferred protection against ischemic kidney injury by reducing oxidative stress, and that upregulation of HIF-2α in the endothelium ameliorated renal damage resulting from ischemia-reperfusion injury. HIFs consist of distinct α-subunits (HIF-1a, HIF-2a, or HIF-3a) and a common β-subunit [42], and are regulated by prolyl-4-hydroxylase domain-containing proteins (PHD1, PHD2, PHD3) via hydroxylation of proline residues on HIF-a subunits. All PHD isoforms have been detected in the kidney and they all regulate the expression of HIF [42], [43]. Previous studies indicated that the actions of the PHDs on different HIF-α isoforms were not equivalent, with PHD2 having greater influence on HIF-1α than HIF-2α. Conversely, PHD3 was found to have more influence on HIF-2α than HIF-1α [42]. Wang et al. [44] demonstrated that inhibition of PHDs prior to renal ischemic reperfusion protected the kidney via stabilization of HIF-1α and -2α and up-regulation of HIF target genes. We recently reported that HIF-1α and HIF-2α could be selectively activated by PHD inhibitor L-mimosine under different physiological conditions [45]. In the present study, we found that Xe pretreatment downregulated PHD1 and upregulated PHD2, but did not affect PHD3 expression in the renal cortex. We speculate that both PHD1 and PHD2 might participate in regulation of HIF-2α. Downregulation of PHD1 initially might activate HIF-2α. A high level of HIF-2α could then increase the activity of PHD2, limiting the up-regulation of HIF-2α. The precise interplay between isoforms of PHD and HIF in Xe exposure and gentamicin nephrotoxicity remains to be elucidated.

miRNA is a recently discovered class of regulatory RNA molecules that regulate gene expression primarily at the post-transcriptional level [46]. miRNAs are known to play an important and ubiquitous role in many vital biological processes including development, cell differentiation, proliferation and apoptosis. Several miRNAs, such as the miR-29 family, miR-382 and miR-21, have been shown to be relevant to renal injury and repair [25], [47]–[49]. miR-21 has been shown to promote proliferation, inhibit apoptosis, and act as a strong anti-apoptotic factor [29], [30], [47]. Our previous work showed that up-regulation of miR-21 contributed to the protection against renal ischemia-reperfusion injury, including renal cell apoptosis, conferred by ischemic preconditioning [49]. In this study, we found that Xe upregulated miR-21, a strong anti-apoptotic factor, in kidney and attenuated tubular cell apoptosis, miR-21 might contribute to Xe-conferred amelioration of gentamicin-induced renal injury.

There are some possible limitations of this gas such as high cost and scarcity, which limite its use to small numbers of experimental and clinical trials, and a complete adverse profile of xenon was unknown because of a limited number of studies. However, the safety and efficiency of xenon anesthesia has been evaluated in large clinical trials [50], and with the development of xenon-closed circuit delivery system and advance in gas extraction technology, xenon dosing and cost would decrease substantially [51]. Considering the cost and its possible toxicity, the use of Xe should be cautious.

In summary, the present study has revealed a novel effect of Xe in protection against gentamicin nephrotoxicity. Further experimental studies and clinical trials on Xe would be valuable in providing further insight into Xe renoprotection and its clinical application.

## Supporting Information

Figure S1 **Experimental protocols 1 (A) and 2 (B).** NS, normal saline; Xe, xenon; N_2_, nitrogen; gent, gentamicin; N_2_+g, nitrogen+gentamicin; Xe+g, xenon+gentamicin; RF, renal function; TUNEL, Terminal deoxynucleotidyl transferase-mediated dUTP nick end labeling; IHC, immunohistochemistry; q-PCR, quantitative polymerase chain reaction; WB, western blot.
(TIF)

## References

[1] Binswanger U (1997) Acute renal failure: changing causes? Kidney Blood Press Res 20: 163. 9293431 10.1159/000174132

[2] Lopez-Novoa JM, Quiros Y, Vicente L, Morales AI, Lopez-Hernandez FJ (2011) New insights into the mechanism of aminoglycoside nephrotoxicity: an integrative point of view. Kidney Int 79: 33–45. 20861826 10.1038/ki.2010.337

[3] Quiros Y, Vicente-Vicente L, Morales AI, López-Novoa JM, López-Hernández FJ (2011) An Integrative Overview on the Mechanisms Underlying the Renal Tubular Cytotoxicity of Gentamicin. Toxicol Sci 119: 245–256. 20829429 10.1093/toxsci/kfq267

[4] Martínez-Salgado C, López-Hernández FJ, López-Novoa JM (2007) Glomerular nephrotoxicity of aminoglycosides. Toxicol Appl Pharmacol 223: 86–98. 17602717 10.1016/j.taap.2007.05.004

[5] El Mouedden M, Laurent G, Mingeot-Leclercq MP, Taper HS, Cumps J, et al (2000) Apoptosis in renal proximal tubules of rats treated with low doses of aminoglycosides. Antimicrob Agents Chemother 44: 665–675. 10681336 10.1128/aac.44.3.665-675.2000 PMC89744

[6] Edwards JR, Diamantakos EA, Peuler JD, Lamar PC, Prozialeck WC (2007) A novel method for the evaluation of proximal tubule epithelial cellular necrosis in the intact rat kidney using ethidium homodimer. BMC Physiol 7: 1–14. 17319948 10.1186/1472-6793-7-1 PMC1810561

[7] Bledsoe G, Crickman S, Mao J, Xia C, Murakami H, et al (2006) Kallikrein/kinin protects against gentamicin-induced nephrotoxicity by inhibition of inflammation and apoptosis. Nephrol Dial Transplant 21: 624–633. 16401625 10.1093/ndt/gfi225

[8] Kalayarasan S, Prabhu PN, Sriram N, Manikandan R, Arumugam M, et al (2009) Diallyl sulfide enhances antioxidants and inhibits inflammation through the activation of Nrf2 against gentamicin-induced nephrotoxicity in Wistar rats. Eur J Pharmacol 606: 162–171. 19374873 10.1016/j.ejphar.2008.12.055

[9] Cuzzocrea S, Mazzon E, Dugo L, Serraino I, Di Paola R, et al (2002) A role for superoxide in gentamicin-mediated nephropathy in rats. Eur J Pharmacol 450: 67–76. 12176111 10.1016/s0014-2999(02)01749-1

[10] Chen LF, Kaye D (2009) Current use for old antibacterial agents: polymyxins, rifamycins, and aminoglycosides. Infect Dis Clin North Am 23: 1053–1075. 19909897 10.1016/j.idc.2009.06.004

[11] Ali BH, Al Za’abi M, Blunden G, Nemmar A (2011) Experimental Gentamicin Nephrotoxicity and Agents that Modify it: A Mini-Review of Recent Research. Basic Clin Pharmacol 109: 225–232. 10.1111/j.1742-7843.2011.00728.x 21599835

[12] Goto T, Suwa K, Uezono S, Ichinose F, Uchiyama M, et al (1998) The blood-gas partition coefficient of xenon may be lower than generally accepted. Br J Anaesth 80: 255–256. 9602599 10.1093/bja/80.2.255

[13] Lachmann B, Armbruster S, Schairer W, Landstra M, Trouwborst A, et al (1990) Safety and efficacy of xenon in routine use as an inhalational anaesthetic. Lancet 335: 1413–1415. 1972207 10.1016/0140-6736(90)91444-f

[14] Rossaint R, Reyle-Hahn M, Schulte Am Esch J, Scholz J, Scherpereel P, et al (2003) Multicenter randomized comparison of the efficacy and safety of xenon and isoflurane in patients undergoing elective surgery. Anesthesiology 98: 6–13. 12502972 10.1097/00000542-200301000-00005

[15] Neukirchen M, Hipp J, Schaefer MS, Brandenburger T, Bauer I, et al (2012) Cardiovascular stability and unchanged muscle sympathetic activity during xenon anaesthesia: role of norepinephrine uptake inhibition. Br J Anaesth 109: 887–896. 22945969 10.1093/bja/aes303

[16] Ma D, Hossain M, Pettet GK, Luo Y, Lim T, et al (2006) Xenon preconditioning reduces brain damage from neonatal asphyxia in rats. J Cereb Blood Flow Metab 26: 199–208. 16034370 10.1038/sj.jcbfm.9600184

[17] Yang T, Zhuang L, Rei Fidalgo AM, Petrides E, Terrando N, et al (2012) Xenon and Sevoflurane Provide Analgesia during Labor and Fetal Brain Protection in a Perinatal Rat Model of Hypoxia-Ischemia. PLoS One 7: e37020. 22615878 10.1371/journal.pone.0037020 PMC3355162

[18] Weber NC, Toma O, Wolter JI, Wirthle NM, Schlack W, et al (2005) Mechanisms of xenon- and isoflurane-induced preconditioning: A potential link to the cytoskeleton via the MAPKAPK -2/HSP27 pathway. Br J Pharmacol 146: 445–455. 16086037 10.1038/sj.bjp.0706324 PMC1576277

[19] Weber NC, Frässdorf J, Ratajczak C, Grueber Y, Schlack W, et al (2008) Xenon induces late cardiac preconditioning in vivo: A role for cyclooxygenase 2? Anesth Analg 107: 1807–1813. 19020121 10.1213/ane.Ob013e31818874bf

[20] Preckel B, Schlack W (2004) Editorial III: Xenon– Cardiovascularly inert? Br J Anaesth 92: 786–789. 15145831 10.1093/bja/aeh137

[21] Ma D, Lim T, Xu J, Tang H, Wan Y, et al (2009) Xenon Preconditioning Protects against Renal Ischemic-Reperfusion Injury via HIF-1α Activation. J Am Soc Nephrol 20: 713–720. 19144758 10.1681/ASN.2008070712 PMC2663824

[22] Yaman I, Balikci E (2010) Protective effects of nigella sativa against gentamicin-induced nephrotoxicity in rats. Exp Toxicol Pathol 62: 183–190. 19398313 10.1016/j.etp.2009.03.006

[23] Karahan I, Ateşşahin A, Yilmaz S, Ceribaşi AO, Sakin F (2005) Protective effect of lycopene on gentamicin-induced oxidative stress and nephrotoxicity in rats. Toxicology 215: 198–204. 16125832 10.1016/j.tox.2005.07.007

[24] Jiang S, Chen Y, Zou J, Xu X, Zhang X, et al (2009) Diverse Effects of ischemic pretreatments on the long-term renal damage induced by ischemia-reperfusion. Am J Nephrol 30: 440–449. 19752532 10.1159/000239574

[25] Liu Y, Taylor NE, Lu L, Usa K, Cowley AW Jr, et al (2010) Renal Medullary MicroRNAs in Dahl Salt-Sensitive Rats : miR-29b Regulates Several Collagens and Related Genes. Hypertension 55: 974–982. 20194304 10.1161/HYPERTENSIONAHA.109.144428 PMC2862728

[26] Yagi K (1998) Simple assay for the level of total lipid peroxides in serum of plasma. Methods Mol Biol 108: 101–106. 9921519 10.1385/0-89603-472-0:101

[27] Appelhoff RJ, Tian YM, Raval RR, Turley H, Harris AL, et al (2004) Differential function of the prolyl hydroxylases PHD1, PHD2, and PHD3 in the regulation of hypoxia-inducible factor. J Biol Chem 279: 38458–38465. 15247232 10.1074/jbc.M406026200

[28] Lieb ME, Menzies K, Moschella MC, Ni R, Taubman MB (2002) Mammalian EGLN genes have distinct patterns of mRNA expression and regulation. Biochem Cell Biol 80: 421–426. 12234095 10.1139/o02-115

[29] Chan JA, Krichevsky AM, Kosik KS (2005) MicroRNA-21 is an antiapoptotic factor in human glioblastoma cells. Cancer Res 65: 6029–6033. 16024602 10.1158/0008-5472.CAN-05-0137

[30] Cheng Y, Zhu P, Yang J, Liu X, Dong S, et al (2010) Ischaemic preconditioning-regulated miR-21 protects heart against ischaemia/reperfusion injury via anti-apoptosis through its target PDCD4. Cardiovasc Res 87: 431–439. 20219857 10.1093/cvr/cvq082 PMC2904662

[31] Rizvi M, Jawad N, Li Y, Vizcaychipi MP, Maze M, et al (2010) Effect of noble gases on oxygen and glucose deprived injury in human tubular kidney cells. Exp Biol Med 235: 886–891. 10.1258/ebm.2010.009366 20472713

[32] Jankauskas SS, Plotnikov EY, Morosanova MA, Pevzner IB, Zorova LD, et al (2012) Mitochondria-targeted antioxidant SkQR1 ameliorates gentamycin-induced renal failure and hearing loss. Biochemistry (Mosc). 77: 666–670. 10.1134/S0006297912060144 22817467

[33] Plotnikov EY, Chupyrkina AA, Jankauskas SS, Pevzner IB, Silachev DN, et al (2011) Mechanisms of nephroprotective effect of mitochondria-targeted antioxidants under rhabdomyolysis and ischemia/reperfusion. Biochim Biophys Acta 1812: 77–86. 20884348 10.1016/j.bbadis.2010.09.008

[34] Silachev DN, Isaev NK, Pevzner IB, Zorova LD, Stelmashook EV (2012) The mitochondria-targeted antioxidants and remote kidney preconditioning ameliorate brain damage through kidney-to-brain cross-talk. PLoS One 7: e51553. 23272118 10.1371/journal.pone.0051553 PMC3522699

[35] Rosenberger C, Mandriota S, Jürgensen JS, Wiesener MS, Hörstrup JH, et al (2002) Expression of Hypoxia-Inducible Factor-1α and -2α in Hypoxic and Ischemic Rat Kidneys. J Am Soc Nephrol 13: 1721–1732. 12089367 10.1097/01.asn.0000017223.49823.2a

[36] Wang Z, Tang L, Zhu Q, Yi F, Zhang F, et al (2011) Hypoxia-inducible factor-1a contributes to the profibrotic action of angiotensin II in renal medullary interstitial cells. Kidney Int 79: 300–310. 20881940 10.1038/ki.2010.326 PMC3107572

[37] Steenhard BM, Freeburg PB, Isom K, Stroganova L, Borza DB, et al (2007) Kidney development and gene expression in the HIF2alpha knockout mouse. Dev Dyn 236: 1115–1125. 17342756 10.1002/dvdy.21106

[38] Scortegagna M, Morris MA, Oktay Y, Bennett M, Garcia JA (2003) The HIF family member EPAS1/HIF-2alpha is required for normal hematopoiesis in mice. Blood 102: 1634–1640. 12750163 10.1182/blood-2003-02-0448

[39] Percy MJ, Furlow PW, Lucas GS, Li X, Lappin TR, et al (2008) A gain-of-function mutation in the HIF2A gene in familial erythrocytosis. N Engl J Med 358: 162–168. 18184961 10.1056/NEJMoa073123 PMC2295209

[40] Paliege A, Rosenberger C, Bondke A, Sciesielski L, Shina A, et al (2010) Hypoxia-inducible factor-2a-expressing interstitial fibroblasts are the only renal cells that express erythropoietin under hypoxia-inducible factor stabilization. Kidney Int 77: 312–318. 20016470 10.1038/ki.2009.460

[41] Kojima I, Tanaka T, Inagi R, Kato H, Yamashita T, et al (2007) Protective role of hypoxia-inducible factor-2alpha against ischemic damage and oxidative stress in the kidney. J Am Soc Nephrol 18: 1218–1226. 17344427 10.1681/ASN.2006060639

[42] Appelhoff RJ, Tian YM, Raval RR, Turley H, Harris AL, et al (2004) Differential function of the prolyl hydroxylases PHD1, PHD2, and PHD3 in the regulation of hypoxia-inducible factor. J Biol Chem 279: 38458–38465. 15247232 10.1074/jbc.M406026200

[43] Li N, Yi F, Sundy CM, Chen L, Hilliker ML, et al (2007) Expression and actions of HIF prolyl-4-hydroxylase in the rat kidneys. Am J Physiol Renal Physiol 292: F207–F216. 16885149 10.1152/ajprenal.00457.2005

[44] Wang Z, Schley G, Türkoglu G, Burzlaff N, Amann KU, et al (2012) The protective effect of prolyl-hydroxylase inhibition against renal ischaemia requires application prior to ischaemia but is superior to EPO treatment. Nephrol Dial Transplant 27: 929–936. 21742784 10.1093/ndt/gfr379

[45] Yu X, Fang Y, Liu H, Zhu J, Zou J, et al (2012) The balance of beneficial and deleterious effects of hypoxia-inducible factor activation by prolyl hydroxylase inhibitor in rat remnant kidney depends on the timing of administration. Nephrol Dial Transplant 0: 1–9. 10.1093/ndt/gfr754 22399494

[46] Liang M, Liu Y, Mladinov D, Cowley AW Jr, Trivedi H, et al (2009) MicroRNA: a new frontier in kidney and blood pressure research. MicroRNA: a new frontier in kidney and blood pressure research. Am J Physiol Renal Physiol 297: F553–F558. 19339633 10.1152/ajprenal.00045.2009 PMC2739705

[47] Godwin JG, Ge X, Stephan K, Jurisch A, Tullius SG, et al (2010) Identification of a microRNA signature of renal ischemia reperfusion injury. Proc Natl Acad Sci U S A. 107 14339–14344. 10.1073/pnas.0912701107 PMC2922548 20651252

[48] Kriegel AJ, Liu Y, Cohen B, Usa K, Liu Y, et al (2012) MiR-382 targeting of kallikrein 5 contributes to renal inner medullary interstitial fibrosis. Physiol Genomics 44: 259–267. 22202692 10.1152/physiolgenomics.00173.2011 PMC3289118

[49] Xu X, Kriegel AJ, Liu Y, Usa K, Mladinov D, et al (2012) Delayed ischemic preconditioning contributes to renal protection by upregulation of miR-21. Kidney Int 82: 1167–1175. 22785173 10.1038/ki.2012.241 PMC3777822

[50] Wappler F, Rossaint R, Baumert J, Scholz J, Tonner PH, et al (2007) Xenon Multicenter Study Research Group: Multicenter randomized comparison of xenon and isoflurane on left ventricular function in patients undergoing elective surgery. Anesthesiology 106: 463–471. 17325504 10.1097/00000542-200703000-00010

[51] Faulkner SD, Downie NA, Mercer CJ, Kerr SA, Sanders RD, et al (2012) A xenon recirculating ventilator for the newborn piglet: developing clinical applications of xenon for neonates. Eur J Anaesthesiol 29: 577–585. 22922476 10.1097/EJA.0b013e3283583c4b

