# Supplementary figures and images for: Intermittent Exposure to Xenon Protects against Gentamicin-Induced Nephrotoxicity
Source: PLoS One. 2013 May 30;8(5):e64329. doi: 10.1371/journal.pone.0064329 (PMC3667819; doi:10.1371/journal.pone.0064329)

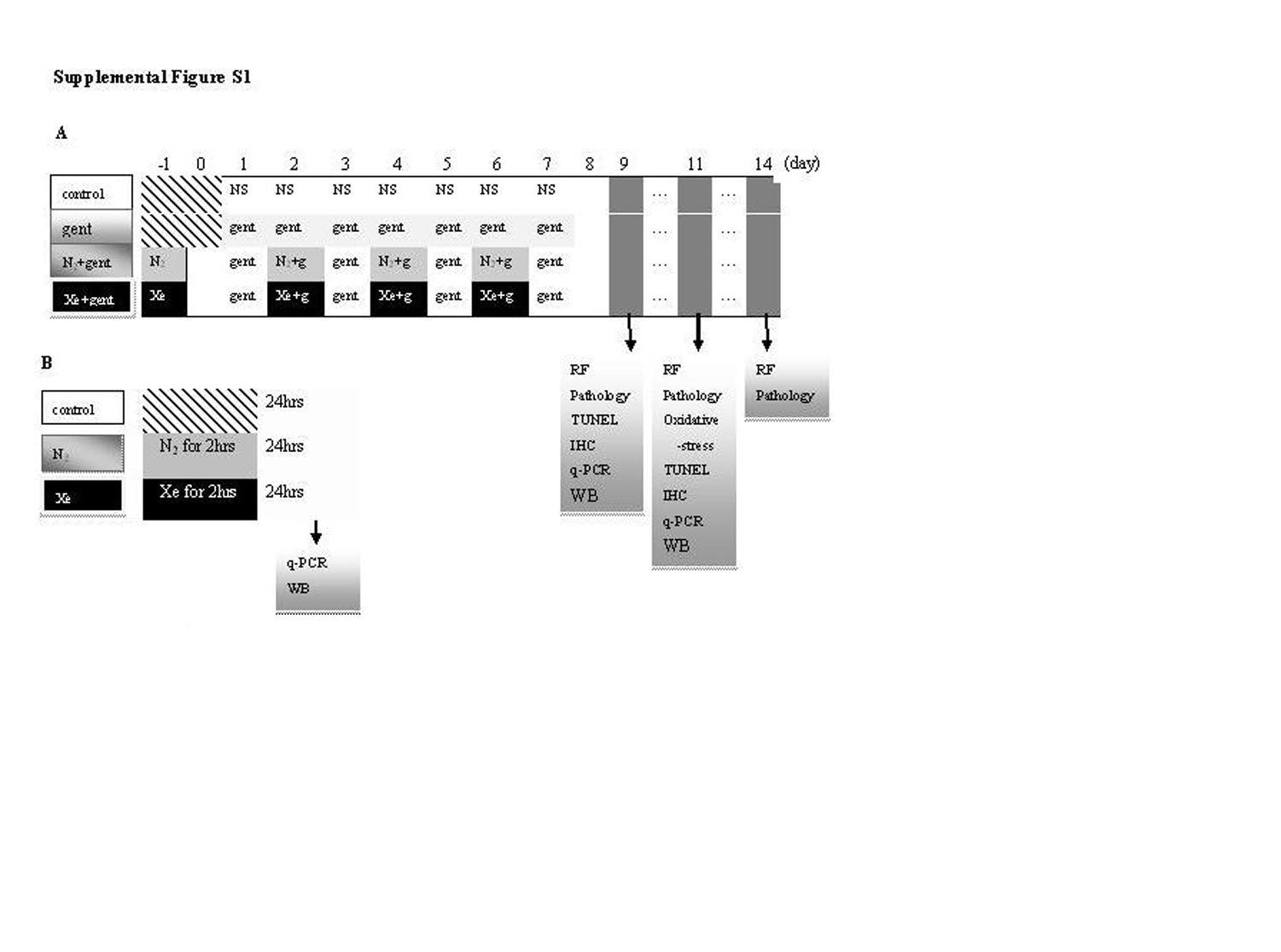

Supplement: Figure S1 — Experimental protocols 1 (A) and 2 (B). NS, normal saline; Xe, xenon; N2, nitrogen; gent, gentamicin; N2+g, nitrogen+gentamicin; Xe+g, xenon+gentamicin; RF, renal function; TUNEL, Terminal deoxynucleotidyl transferase-mediated dUTP nick end labeling; IHC, immunohistochemistry; q-PCR, quantitative polymerase chain reaction; WB, western blot. (TIF) [file pone.0064329.s001.tif]
